# Supplementary material for: Evaluating [18F]FDG and [18F]FLT Radiotracers as Biomarkers of Response for Combined Therapy Outcome in Triple-Negative and Estrogen-Receptor-Positive Breast Cancer Models
Source: Int J Mol Sci. 2023 Sep 15;24(18):14124. doi: 10.3390/ijms241814124 (PMC10532149; doi:10.3390/ijms241814124)
Supplement: Supplementary file 1 [file ijms-24-14124-s001.zip › ijms-2517957-supplementary.pdf]

International Journal of
Molecular Sciences

Supplementary Materials

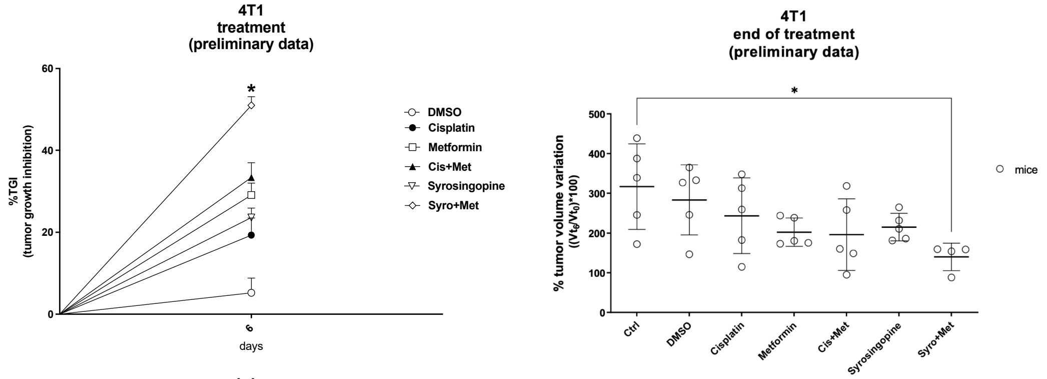

(a) **(b)**

Figure S1. Treatment efficacy results on TNBC model (4T1 cell line), expressed as **(a)** percentage of tumor growth inhibition (%TGI) compared to the control group, calculated at different time points. Mice were randomly assigned to six groups of treatment: control (n. 5); DMSO (n. 5); cisplatin (n. 5, dose 3 mg/kg i.p., twice a week); MET (n. 5, dose 250 mg/kg i.p., daily); cisplatin plus MET (n. 5); SYRO (n. 5, dose 7.5 mg/kg i.p., three times per week) and SYRO plus MET (n. 4). *(Tumor growth inhibition >49% is considered meaningful). Data at the end of treatment (6 days), were even expressed as **(b)** percentage of tumor volume variation compared to the starting of treatment. Average values calculated per group ± SD, (Student's t-test; *$p < 0.05$ vs. vehicle group).

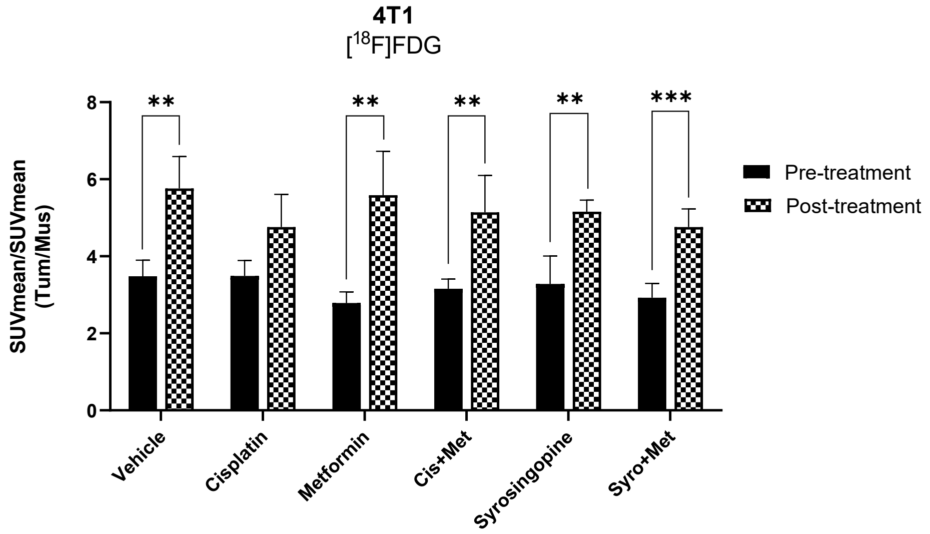

(a)

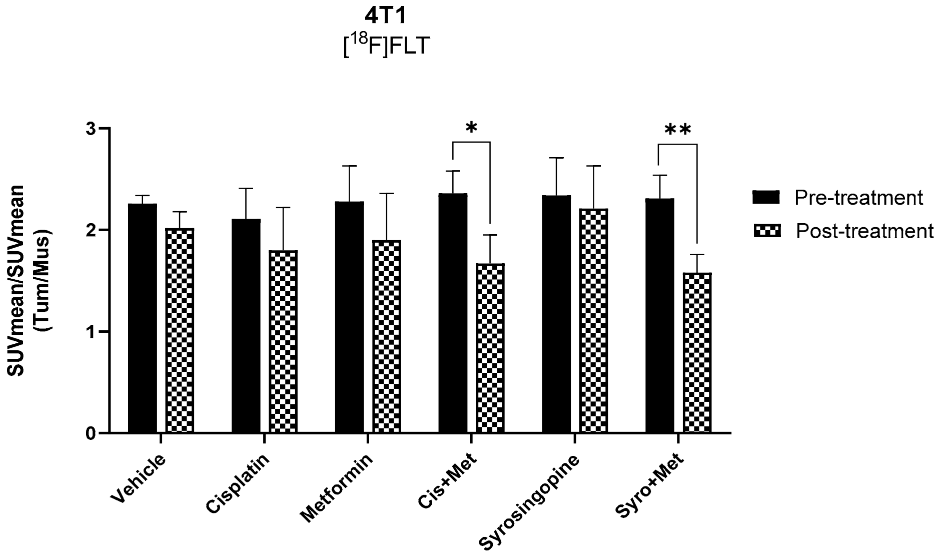

(b)

Figure S2. 4T1 tumors-bearing balb/c mice (vehicle (ctrl): n. 5; cisplatin: n. 4; metformin: n. 5; cisplatin plus metformin: n. 5; syrosingopine: n. 5 and syrosingopine plus metformin: n. 3) were injected i.v. with either **(a)** [^{18}F]FDG or **(b)** [^{18}F]FLT radiotracer (~4.7 MBq/mouse). Radiotracer uptake was assessed for each experimental group at 60 minutes post injection by whole-body PET/CT acquisitions performed pre-treatment and post-treatment. The quantification data are reported as tumor to muscle ratios of SUV mean values. Bars, mean ± SD (Student's t-test; *p <0.05, **p <0.01 and ***p <0.01 vs. post treatment).

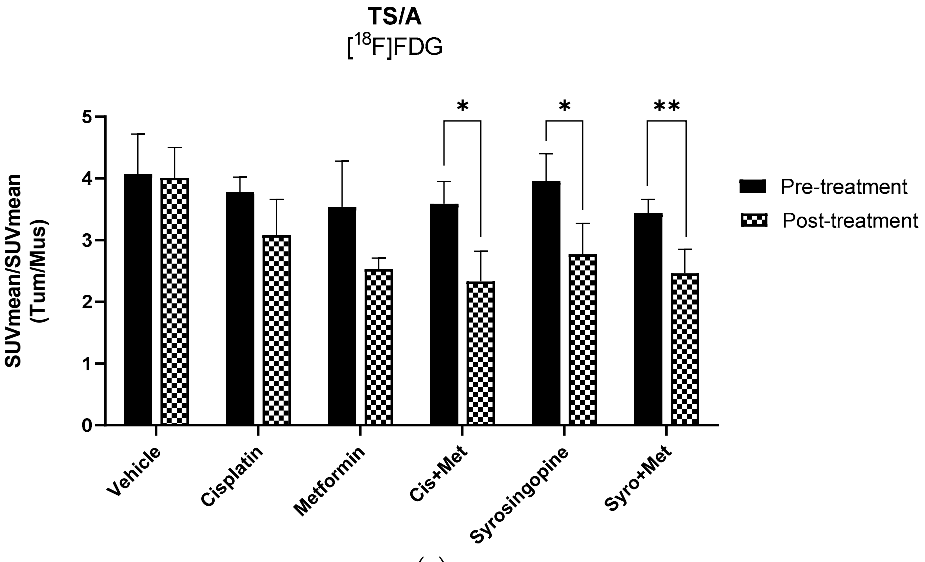

(**a**)

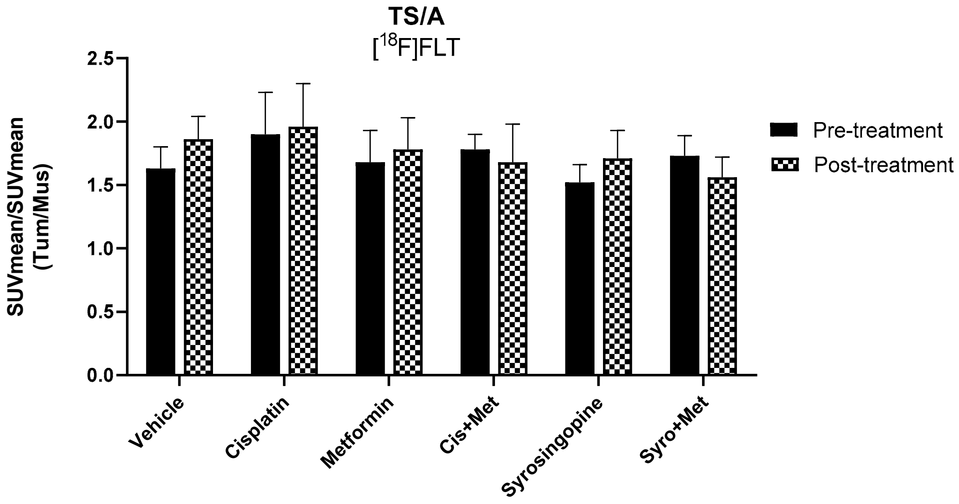

(**b**)

Figure S3. TS/A tumors-bearing balb/c mice (n=5 mice per group) were injected i.v. with either **(a)** [18F]FDG or **(b)** [18F]FLT (bottom) radiotracer (~4.7 MBq/mouse). Radiotracer uptake was assessed for each experimental group at 60 minutes post injection by whole-body PET/CT acquisitions performed pre-treatment and post-treatment. The quantification data are reported as tumor to muscle ratios of SUV mean values. Bars, mean ± SD (Student's t-test; *p <0.05 and **p <0.01 vs. post treatment).

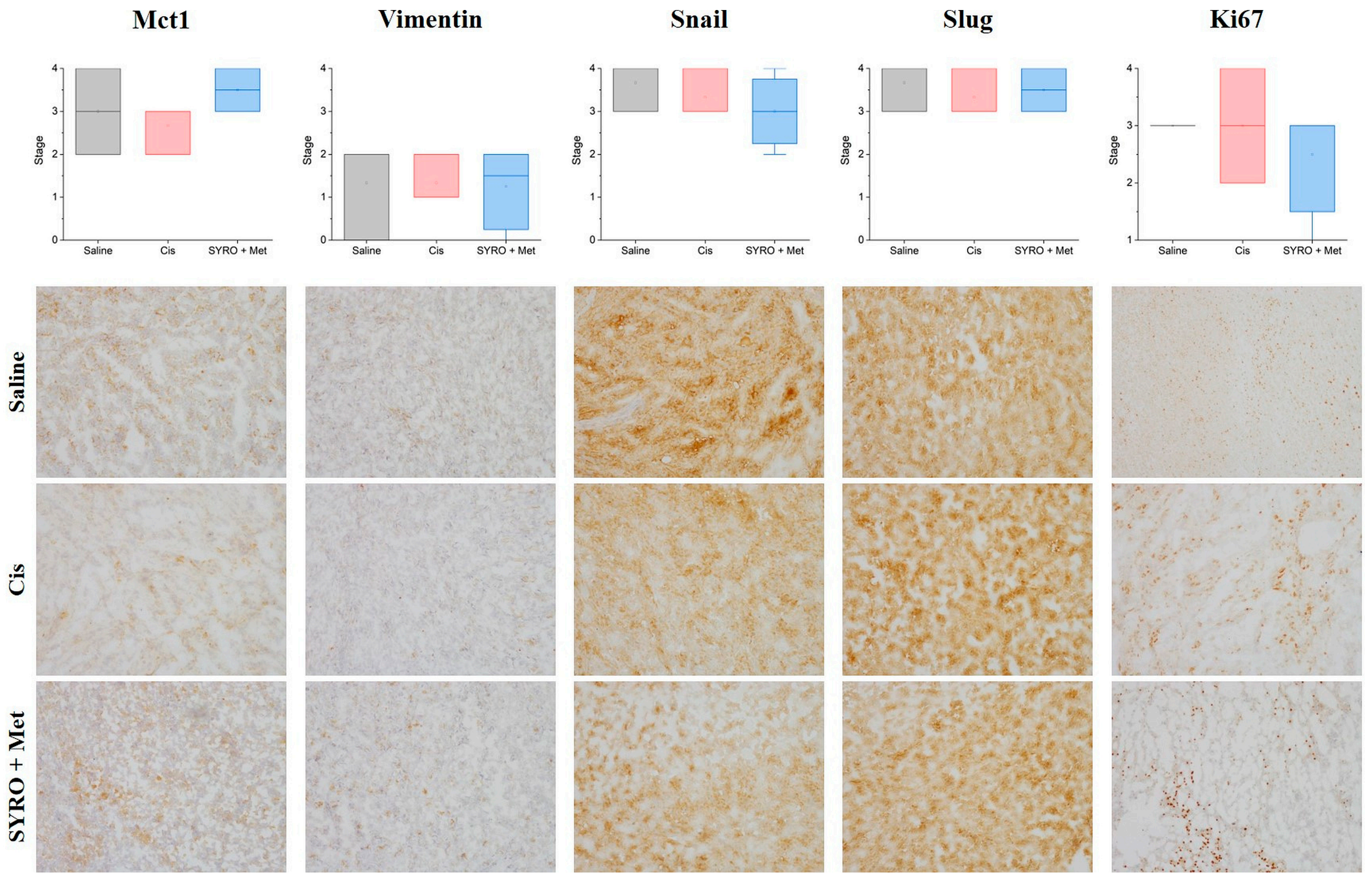

Figure S4. Immunohistochemical evaluation of Mct1 lactate/H+ transporter, the EMT-related marker *Vimentin*, *Snail* and *Slug*, and *Ki67* as index of proliferation. Extent of expression was semi-quantitative scored and results were represented by whiskers and box plots. On the bottom, representative micrographs showing the extent of expression for *Mct1, Vimentin, Snail, Slug* and *Ki67* in tumor samples obtained from xenotransplant mice treated with saline (ctrl), cisplatin (cis) and Syrosingopine plus metformin. Statistical analysis: one-way Analysis of Variance (ANOVA) test; all = not significant. Original magnification: 10x.

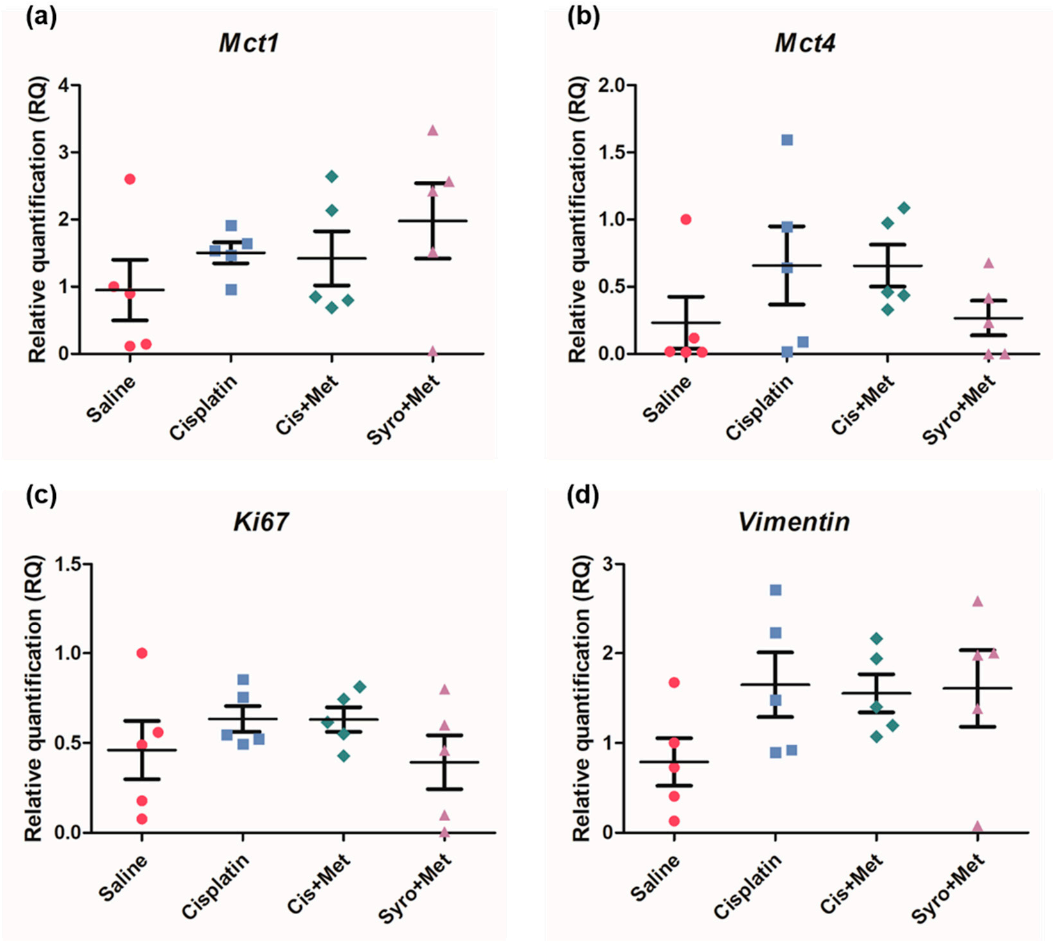
(a)
Mct1
Relative quantification (RQ)
0
1
2
3
4
Saline
Cisplatin
Cis+Met
Syro+Met
(b)
Mct4
Relative quantification (RQ)
0.0
0.5
1.0
1.5
2.0
Saline
Cisplatin
Cis+Met
Syro+Met
(c)
Ki67
Relative quantification (RQ)
0.0
0.5
1.0
1.5
Saline
Cisplatin
Cis+Met
Syro+Met
(d)
Vimentin
Relative quantification (RQ)
0
1
2
3
Saline
Cisplatin
Cis+Met
Syro+Met

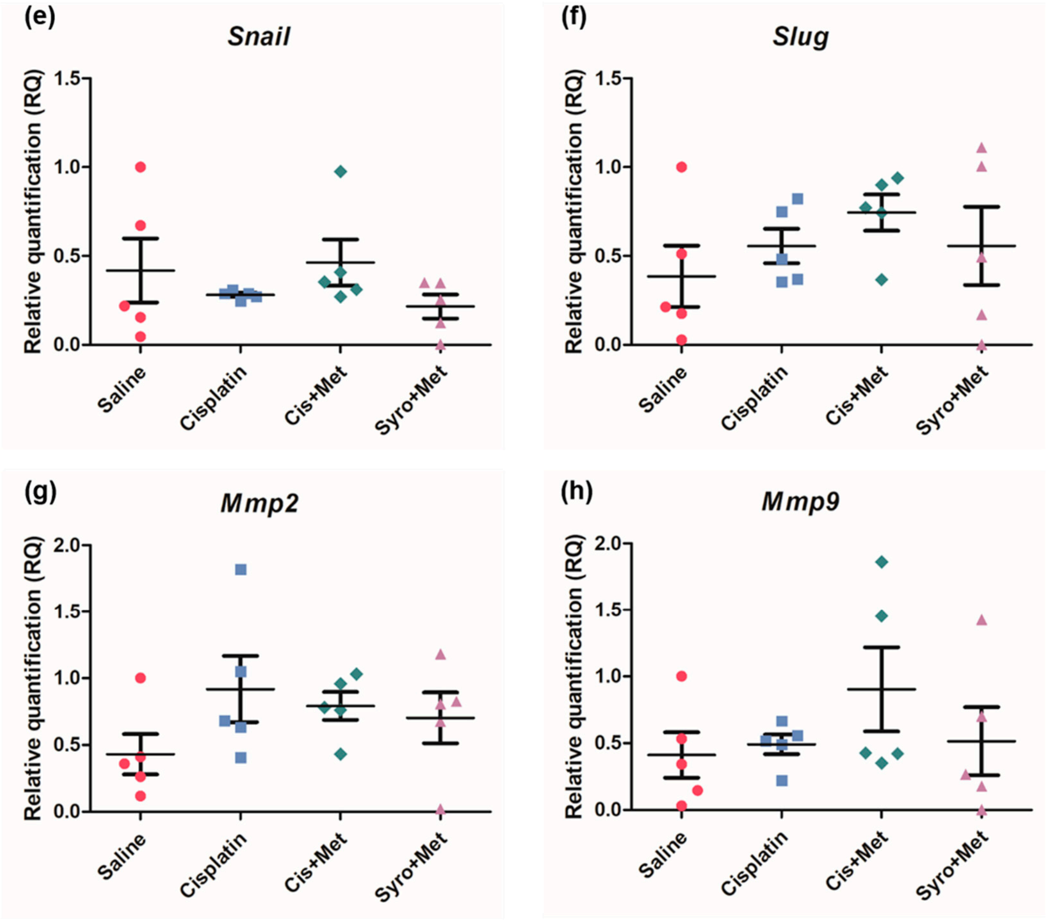

Figure S5. Expression levels of *Mct1* (**a**), *Mct4* (**b**), *Ki67* (**c**), *Vimentin* (**d**), *Snail* (**e**), *Slug* (**f**), *Mmp2* (**g**) and *Mmp9* (**h**) in tissue from 4TS/A tumors-bearing balb/c mice (saline (ctrl): n. 5; cisplatin: n. 5; cisplatin plus metformin: n. 5 and syrosingopine plus metformin: n.5). Data are expressed as fold increase ± SEM (Student's t-test vs. saline group).
